# Lime Salts Necessary to Children

**Published:** 1873-05

**Authors:** D. C. Hawxhurst


### DENTAL REGISTER.

Vol. XXVII.] MAY, 1873. [No. 5.

### LIME SALTS NECESSARY TO CHILDREN.

BY D. C. HAWXHURST.

There are few respectable practitioners of dentistry at the present time, who do not know the important relation which exists between the mineral salts of food, and the proper organization and development of the teeth of children. Much attention has been given, and much more must in the future be given to this subject. Through all our recent literature are scattered articles on the "Lime Salts," "The Phosphates," "Early Development of Teeth Dependent upon a Supply of their Mineral Constituents," "Calcification of Growing Teeth," etc.

The principle which runs throughout all this writing, and constantly glows and brightens as it is the more examined, is that *one of the fundamental conditions of normal organization of developing teeth, is a liberal supply of mineral salts.*

As in raising wheat and barley we must supply to the soil, if it lacks them, those mineral substances which help to make up the substance of these grains; so in watching over the development of children's teeth we must make ourselves certain beyond a doubt that the blood furnished them contains not alone the elements but the proximate elements necessary to the up-building of normal tooth-structure.

There is no appeal; these elements must be supplied to the teeth. Otherwise the minute constitution of their tissues will be defective, and serious forms of dental disease are likely to result. The attempt to keep bees on a sandy plain where there are no flowers, or an effort to draw profit from a poultry yard without supplying lime salts for the egg-shells, could not result in more utter failure, than the equally absurd and impossible enterprise of furnishing a child with dense and perfect teeth without giving it proper foods.

Parents may as well recognise first as last that every growing child must be supplied in its daily food with the mineral salts that are to form the basis of its teeth. Those who have children in charge should understand that if this supply is not made these organs will contain defects of structure that must encourage early decay; that they will in short be made up of molecules, every one of which is impotent to resist the action of even comparatively mild morbific agents.

In the food of most children, whose parents have not given special attention to the subject, there is but a scanty supply of the mineral constituents of tooth and bone; this is no fault of nature herself. The raw material before it is fitted by art for its place on our tables, contains all the elements required in the nourishment of the teeth.

This is true of wheat, which in this country is one of our principles sources of mineral food. Ground whole and unbolted it makes a wholesome, nutritious bread, rich in those mineral salts which are necessary to give density to the teeth. Furnished with an abundance of such bread, the blood of the growing child, whose digestive organs are healthy, can not fail to be well supplied with the mineral constituents of teeth; and unless there were some powerful causes acting to oppose assimilation, the dental tissues under such circumstances must be well calcified.

But when flour is bolted, it is largely deprived of its mineral salts. They have adhered to the gluten nearest the bran or husk, and have passed into the bran bins of the mill. When it is remembered that in this country, wheat bread is our standard source of phosphatic salts, and our mode of preparing it is such as to rob it of much of these salts, we can no longer remain blind to the cause which is producing so many poorly organized teeth.

But scantily furnished with lime salts throughout the whole period of their growth and formation, they present the gravest defects of structure. Even to the unaided senses, and without the application of any more delicate tests, they appear to be lacking in density and tenacity of substance. They cut easily under the excavator or drill, and their tissues compare with those of well-organized teeth, as the wood of pine compares with that of seasoned hickory.

On making sections the pulps are found to be large, the apical foramina expanded, and the roots soft.

Under microscopic examination the tubuli of the dentine are large. Under chemical examination the relative proportion of mineral matter is small. Not enough of the phosphatic salts have been infiltrated into the organic matrices. The whole structure of the tooth by its softness, by the animal nature of its tissues, by the state of development in pulp, foramina and tubuli, gives evidence of insufficient calcification. And this has no doubt resulted from a scanty supply of mineral salts in the food, while the organ was undergoing the process of development.

That such teeth should decay is not a matter of surprise. Their delicate structures waste away visibly from month to month under the influence of oxygen and the disintegrating forces at work in the mouth. The deciduous teeth suffer extensive injury soon after their eruption; and the first permanent molars, in many cases, scarcely burst the gum, and make their way into the open cavity of the mouth before decaying cavities burrow into their substance. These results are entirely unnatural, and though hastened by neglect, and aggravated by the reckless use of improper aliments, are at bottom due to the defects and imperfections of the decaying tissues.

Here then is one important fact in the solution of the problem of *caries*. We must secure greater *integrity of structure* in the decaying tissue. And among other conditions tending to this end, will be found the furnishing of the patient from the earliest period of his existence, with nutritive supplies that are rich in the mineral constituents of teeth. This will be done at first through the mother, and at a later period by direct feeding.

The phosphatic salts are abundant in several common articles of diet. Wheat, barley, beefsteak and fish, may be relied upon if their chemical composition is not interfered with during preparation for the table. Oatmeal, Southern corn, oysters, peas, beans, and milk, are all rich in phosphatic salts. They should be liberally supplied to the mother during gestation and lactation, and to the child during infancy and youth.

There are a multitude of questions connected with this subject which I have made no attempt to answer. Some of these relate to the non-assimulation of the salts of food during anæmia; the necessity for a more liberal supply during gestation and lactation than is easily obtained from common foods; the best treatment of plants designed for food, to increase the amount of mineral salts which they contain; how to induce the stomach to extract the greatest amount from foods; how to render artificial preparations of the mineral salts digestible; under what circuustances will they be useful to the patient. These are some of the questions which ought to be answered by those who know.

